# Risk-stratified optimization of skin care protocols for children with cardiac monitors: a data-driven clinical framework

**DOI:** 10.3389/fped.2025.1618405

**Published:** 2025-08-28

**Authors:** Kun Zhang, Ning Chen, Hui Yuan

**Affiliations:** ^1^ Department of Pediatric Cardiology, Shandong Provincial Hospital Affiliated to Shandong First Medical University, Jinan, China; ^2^ Department of Digestive Endoscopy Center, Shandong Provincial Hospital Affiliated to Shandong First Medical University, Jinan, China

**Keywords:** cardiac monitoring, children, medical adhesive-related skin injury, risk factors, nursing care

## Abstract

**Objective:**

To develop a risk-stratified nursing protocol reducing electrode-related skin injuries (MARSI) in pediatric cardiac monitoring.

**Methods:**

Using logistic regression, we analyzed risk factors (allergy history, eosinophil indices, wear duration) in 229 control-group children (June to August 2023) receiving standard care. Derived protocol integrated risk thresholds. Validation involved 183 intervention-group children (September to December 2023).

**Results:**

Skin injury correlated negatively with age/weight/height/BMI (*p* *<* 0.05) and positively with allergy history/wear time/eosinophils (*p* *<* 0.05). The intervention group showed significantly lower MARSI incidence (15.8% vs. 40.6%, *p* *<* 0.001), with reduced redness (4.2% vs. 32.8%) and rashes (2.1% vs. 9.2%) (*p* *<* 0.05).

**Conclusion:**

This risk-stratified nursing protocol reduces pediatric MARSI incidence, supporting clinical implementation.

## 1 Introduction

Since the mid-to-late 20th century, the introduction of bedside electrocardiogram (ECG) monitoring devices has signified a major advance in the continuous monitoring of patients with cardiovascular diseases. Following the introduction of the first ECG monitor in China in 1928, ECG monitoring has become integral to modern clinical nursing. Functioning as the critical physical interface between monitoring devices and patients (particularly for pediatric patients with sensitive skin), the electrode patch continuously acquires bioelectrical signals through the integrated components, including non-woven or cellulose acetate fabric backings, electrode clips, electrode cores, and highly adhesive medical-grade hydrogel matrices. These patches maintain continuous skin adhesion for extended durations, sometimes remaining attached for several consecutive days.

However, this physical interface inherently carries risks of skin injury. Specifically, conventional non-woven or foam electrode patches exhibit limited breathability, which impedes normal cutaneous respiration and sweat evaporation. In combination with the adhesive properties of the conductive hydrogel, these factors may provoke various cutaneous irritation reactions. Upon patch removal, the affected skin area may present erythematous rashes with pruritus, and in some instances, develop microvesicles. More severe cases may include localized epidermal denudation with pain. These clinical manifestations represent adhesive-related dermatologic complications.

The International Society for Dermatologic and Ostomy Care defines such injuries as Medical Adhesive-Related Skin Injury (MARSI), characterized by pathological changes such as erythema, blistering, epidermal exfoliation, or maceration persisting in the patch area for 30 min after the removal of the medical adhesive (1).

Its incidence demands attention: MARSI affects approximately 7%–15% of adult inpatients (2). In pediatric patients, the severity and particularity of this issue are prominent. Children, especially infants, possess developmentally immature skin barriers. Their stratum corneum (SC) is thin, and its integrity remains fragile. A study demonstrates that the mean thickness of the SC on the thighs of East Asian infants and adults measures 5.3 ± 1.4 μm and 7.9 ± 1.8 μm, respectively (3). This thin barrier, combined with high skin water content and a large body surface area (BSA) ratio, markedly weakens barrier function (4). Consequently, children exhibit heightened sensitivity to adhesive irritation, occlusion, and friction, resulting in elevated MARSI incidence rates of 23.53% to 54.17% (mean 37.15%) (5). For example, a pediatric cardiac surgery patient who requires long-term postoperative monitoring is likely to develop obvious erythema and effacement of skin markings (pre-impaction manifestation) at the electrode attachment sites on the chest and abdomen after 24–48 h of monitoring. This prolonged application leads to a significant risk of epidermal damage.

Studies have identified electrode patches as a primary “culprit” that trigger MARSI (6). These seemingly minor skin injuries carry significant clinical consequences. They directly cause pruritus and pain in children and may also lead to secondary bacterial infections (e.g., when Staphylococcus aureus or Streptococcus invades the damaged skin), such complications not only exacerbate the suffering of pediatric patients but may also significantly prolong hospitalization, increase additional antibacterial drug use and wound treatment costs, and ultimately increase both healthcare system burden and familial economic strain. A study revealed that children with skin injuries and infections caused by electrode patches require enhanced antibiotic treatment therapy and wound care, prolonging anticipated discharge by 3–4 days. Current research on MARSI pathogenesis mainly focuses on three aspects: the patient's intrinsic pathological characteristics (e.g., a history of skin allergies or eczema); clinical practice (e.g., whether the patch is removed gently) and drug-related effects (e.g., the weakening of the skin barrier by the use of glucocorticoids) (7, 8). However, in ECG monitoring, a specific, frequently encountered clinical scenario that typically involves long-term continuous use, a systematic and in-depth exploration of key factors remains lacking to date. Furthermore, China lacks standardized clinical skin care practice guidelines specifically for pediatric patients who require long-term ECG monitoring (more than 48 h). Existing interventions, including the application of hydrocolloid dressings to blistered or rash areas, and the pretreatment of skin with liquid barrier films or skin protectants, can mitigate injuries in some cases (9), but their high economic costs and complex operations restrict widespread adoption and compliance, underscoring the need for further optimization.

This research is expected to ultimately improve the skin health in pediatric patients during ECG monitoring, reduce iatrogenic skin injury rates, enhance care quality and patient comfort, and alleviate potential healthcare burdens. To achieve this goal, the study enrolled 412 pediatric patients. All required ECG monitoring in the pediatric cardiology department of our hospital from June 2023 to December 2023. The study will evaluate the safety, feasibility and effectiveness of the new skin care plan designed based on the above approach. With the expectation of optimizing the skin care practices, we aim to provide important scientific evidence and practical guidance for children requiring long-term ECG monitoring.

## 2 Objects and methods

### 2.1 Subject of the study

A total of 229 children who were hospitalized in the Department of Pediatric Cardiology at our hospital and required ECG monitoring from June to August 2023 were selected as the control group. Additionally, 183 children who were hospitalized in the same department and required ECG monitoring for disease treatment from September to December 2023, were selected as the intervention group.

Inclusion criteria for the study included children aged ≤14 years, with cardiac monitoring for ≥48 h, intact skin without breakage or redness prior to monitoring, and no history of allergic reactions to medical adhesive tape.

Exclusion criteria included children aged >14 years, with monitoring for ≤48 h, uncooperative families, damaged skin before monitoring, or a history of allergy to medical adhesive tape. Children requiring resuscitation, such as those with cardiogenic shock, cardiac arrest, or ventricular fibrillation, were also excluded. The study was reviewed and approved by the Ethics Committee of the Provincial Hospital of Shandong First Medical University, SWYX: NO. 2024-164.

### 2.2 Research methodology

#### 2.2.1 Research design and data collection

**Figure 1:**
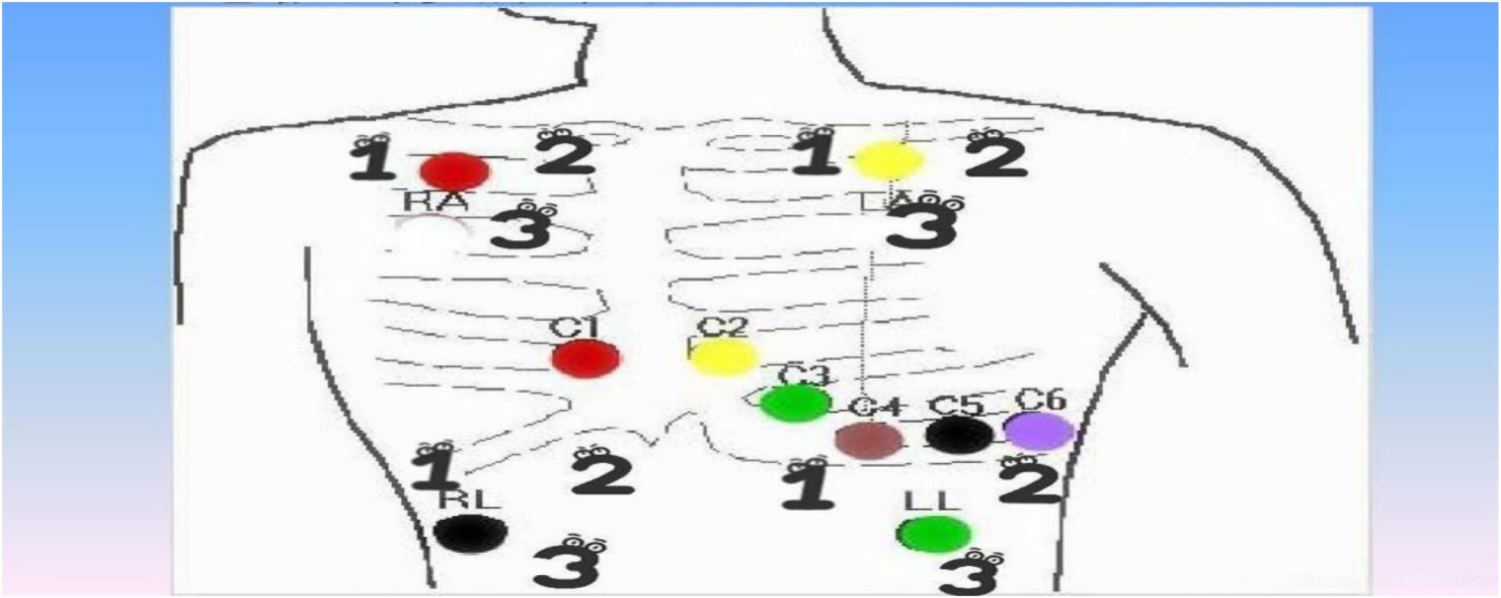
Electrode patch rotation mapping.

This study adopts a prospective observational design, based on the MARSI diagnostic criteria (10), constructs a three-in-one programme of “biomarker early warning-precision protection-intelligent quality control”, innovatively incorporates the absolute value of EOS into the predictive index, which compensates for the lack of laboratory early warning indexes in the existing guideline, and at the same time, by adopting the layered protection strategy, the protection effect has been significantly enhanced. Implemented closed-loop management through the PDCA electronic platform. Identified high-risk individuals based on risk factors, distinguishing from the traditional universal protection, thereby improving clinical efficiency and reducing healthcare costs. The specific scheme is as follows:

(1) Control group nursing protocol:
  (a) Routine operation: The skin was cleaned with water, and the electrode patch was applied. The patch and its position were changed every 48 h.
  (b) Removal method: The patch was slowly removed, or moistened with sterile saline before removal.
  (c) Injury treatment: Local disinfection was performed, followed by the application of mupirocin ointment.
(2) Intervention group care plan (based on the risk prediction model). Multiple regression analysis identified age, BMI, allergy history, wearing time, and eosinophil (EOS) level as independent predictors of MARSI (*p* *<* *0.05*, Exhibit 1). Stratified protection strategies were developed based on these predictors:
  (a) High-risk children (age <3 years, BMI <15 kg/m^2^, or absolute EOS >0.5 × 10^9^/L): A thin silicone foam dressing (3M Tegadery) is pre-positioned underneath the electrode pads prior to wear for barrier protection (11). The patch was moisturised with liquid paraffin for 5 min prior to removal and then slowly removed (1). Stabilisation phase with intermittent suspension of monitoring (minimum of 4 h off every 48 h).
  (b) Routine monitoring of children: The local skin is evenly coated with non-allergic emollient cream (Cetaphil) before sticking, and wait for 10 min for absorption; according to the preset “electrode patch rotation chart” (Figure 1), the replacement position is more than 5 cm away from the removal position, which not only facilitates the nurses on duty to choose the replacement position according to the chart, avoiding the vulnerable area (24-hour ECG sticking area), but also increases the local skin repair time of the children who have been wearing the monitor for a long period of time. Repair time.
(3) Data collection and assessment:
  (a) Skin scoring: Thirty minutes after the electrode patch was removed, the resulting skin condition was scored as follows: 0 for intact, 1 for redness, 2 for rash, 3 for blisters, and 4 for breakage. The degree of injury was recorded on a four-level scale.
  (b) Laboratory metrics: The percentage and absolute values of EOS were collected for the baseline period (Sysmex XN-1000 Hematology Analyzer).
  (c) Quality control: Two blinded nursing specialists independently scored the data (Kappa consistency coefficient = 0.89).

#### 2.2.2 Nursing process standardization

(a) Operational training: Three-stage training, including case simulation, theoretical lectures, and practical assessments, ensured that nurses mastered MARSI risk assessment and protection techniques (100% pass rate in the assessment).
(b) Time reminder system: An intelligent bedside timer (XiaoMi DOL-288) automatically reminded staff when to replace the electrode patch, eliminating the need for manual card records.
(c) Adhesive residue treatment: Baby Emollient oil (Mustela) was gently used to wipe off residue, while alcohol-based solvents were prohibited.

#### 2.2.3 Statistical analysis

Data were analyzed using *SPSS* 26.0. Continuous variables were expressed as mean ± standard deviation, and categorical variables were described as frequency (%). Comparisons between groups were made using the independent samples *t*-test or Mann–Whitney *U*-test. Multifactorial analysis was performed using the logistic regression model (*α* = 0.05). Data analysis was performed using SPSS 26.0. Continuous variables were expressed as mean ± standard deviation, while categorical variables were presented as case frequencies (%). Group comparisons employed independent sample *t*-test or the Mann–Whitney *U*-test. Multivariate analysis utilized the logistic regression model (*α* = 0.05). SPSS 26.0 was selected specifically for its mixed-effects model module capable of processing hierarchically nested data. Multivariate logistic regression was implemented to construct the MARSI prediction model. This approach offered distinct advantages for analyzing heterogeneous data, as it allowed simultaneous assessment of mixed predictors such as continuous variables (e.g., age) and categorical variables (e.g., allergy history). Therefore, we chose logistic regression instead of machine learning models, because the sample size (*n* = 412) of this study did not meet the threshold for training complex models.

#### 2.2.4 Strengths and weaknesses of the methods

The protocol innovatively integrates objective biomarkers (e.g., eosinophil indices) to stratify MARSI risk and demonstrating the potential of biomarker-guided nursing. Three limitations require attention:

(a) Generalizability constraints: The single tertiary-center cohort lacks diversity and primary-care validation;
(b) Unquantified resource burden: Marginal costs of additional testing (e.g., eosinophil monitoring) remain unmeasured;
(c) Sustainability uncertainty: A 4-month follow-up is insufficient to evaluate the impact of seasonal allergies.

## 3 Quality control and implementation supervision

A “Plan-Do-Check-Amend (PDCA)” closed-loop management system was established to ensure comprehensive quality control of the improved care program.

(a) Daily supervision: The quality control team verified the nursing staff's compliance with risk assessments (age/BMI/allergy history), electrode patch rotation standardization, and MARSI protective measure implementation daily.
(b) Weekly special review: The project team reviewed the completeness of skin status records and nursing logs for high-risk cases weekly.
(c) Monthly efficacy assessment: The head nurse led monthly meetings to adjust the quality control focus, combining the trend of skin injury incidence with the operation pass rate (≥95% of preset criteria).
(d) Data-driven continuous improvement: An electronic adverse event reporting system (nursing management platform) was established to track MARSI events in real time. The Revised Guidelines for Electrode Patch Operation SOPs were developed based on aggregated data, focusing on optimizing the adhesive removal process and the suspension of supervision in high-risk children.

## 4 Endpoint indicators and assessment methods

### 4.1 Primary endpoint

The final effect was comprehensively evaluated through three dimensions: effectiveness, safety, and feasibility.

(a) Effectiveness evaluation: The cumulative MARSI incidence and injury severity before the baseline period (3 months) and after the intervention period (4 months) were compared. A digital four-level grading standard was utilized to reduce single assessment time while improving accuracy.
(b) Safety evaluation: The evaluation indicators were the secondary infection rates and the changes in pain index based on FLACC scale scores.
(c) Feasibility evaluation: The evaluation indicators were the nurse's operation compliance rates and the time for a single operation, including both electrode patch replacement and skin pre-treatment time.

### 4.2 Secondary endpoints

Time to skin repair (days from discovery of damage to epidermal integrity).

## 5 Results

### 5.1 Comparison of baseline data between the Two groups

Among the 412 children, 229 (55.6%) were assigned to the conventional care program and 183 (44.4%) to the modified care program. In the conventional care group, there were 107 males and 122 females, aged 0–14 years, with a mean age of 6.33 ± 4.25 years. Their weights ranged from 2.5 to 122.0 kg, with a mean weight of 29.37 ± 19.78 kg. In the modified care group, there were 109 males and 74 females, aged 1–14 years, with a mean age of 6.09 ± 3.90 years. Their weights ranged from 3.7 to 101.0 kg, with a mean weight of 26.85 ± 18.38 kg. Comparison of baseline data, such as age, weight, height, wearable supervision duration, and allergy history, between the two groups showed no statistically significant differences (all *P* > 0.05). However, the BMI of children in the conventional care group was higher than that of the modified care group, and this difference was statistically significant (*P* < 0.05), as shown in Table 1.

**Table 1:** Comparison of baseline data between the two groups.

Variant	Control subjects	Intervention group	Statistical value	*P*
Age (years)	6.33 ± 4.25	6.09 ± 3.90	−0.595	0.552
Genders				
Boy (%)	107 (46.7)	109 (50.2)	−2.697	0.007*
Girl (%)	122 (53.3)	74 (40.2)		
Weight (kg)	29.37 ± 19.78	26.85 ± 18.38	−1.333	0.183
Height (cm)	121.61 ± 30.98	120.04 ± 30.21	−0.517	0.605
BMI	17.55 ± 5.08	16.57 ± 4.01	−2.117	0.035
Hours of cardiac monitoring utilized (h)				
48–144	188 (82.1)	130 (71.0)	1.203	0.23
145–288	32 (14.0)	35 (19.1)	−0.178	0.859
>288	9 (3.9)	18 (9.9)	0.304	0.764
Have a history of allergies (%)	34 (14.8)	23 (12.6)	−0.128	0.899
Percentage of eosinophils	3.29 ± 2.94	2.81 ± 3.29	1.542	0.124
Absolute eosinophils	0.26 ± 0.31	0.21 ± 0.24	1.821	0.069

* Denotes *P* < 0.01.

### 5.2 Analysis of factors affecting skin damage in children

Statistical analysis of clinical data for children admitted to the Pediatric Cardiology Department of our hospital from June to August 2023 revealed that 229 children wore cardiac monitors to track heart rate and rhythm changes. Of these, 93 developed local skin problems after electrode patch removal, including redness, rash, blisters, and ulceration, as shown in Table 2. Among these children, 39 had a history of allergic diseases, 13 had allergic conditions, 15 had a history of drug allergies, 11 had a history of food allergies, 1 had a history of medicine allergy, and 2 had a history of allergic reactions to anti-allergic drugs. Analysis of the relevant factors leading to skin damage concluded that age, weight, height, BMI, and total skin damage score were negatively correlated, while the history of allergies, duration of wear, eosinophil percentage before wearing the monitor, and eosinophil absolute value were positively correlated with skin damage. No correlation was found between gender and total skin damage score. Further risk factor analysis was performed to assess the coefficient of determination and regression degree of each factor to determine the main risk factors.

**Table 2:** Analysis of skin lesions in cardiac monitoring from June to August 2023.

Items	Total cases (*n* = 229)	Number of cases per month (*n*/month)	Percentage (%)
Redness of the skin	53	17.67	56.99
Rashes	29	9.67	31.18
Blisters	7	2.33	7.53
Breakages	4	1.3	4.3
Add up the total	93	25.3	100

#### 5.2.1 Basic characterization and one-way analysis of related indicators

The 229 children were divided into two groups based on the presence of skin lesions: the skin lesion group (*n* = 62) and the non-skin lesion group (*n* = 167). Eight variables potentially associated with skin lesions were analyzed using one-way logistic regression. Seven candidate variables were initially identified as significant (*P* < 0.01): age, height, BMI, allergy history, eosinophil percentage, eosinophil absolute value, and duration of wearable supervision, as shown in Table 3.

**Table 3:** Univariate analysis of basic characteristics and related indicators of children with and without skin lesions.

Variant	Group with skin lesions	Group without skin lesions	*β*-value	Standard error	Wald	*P*	Or value (95% CI)
	(*n* = 62)	(*n* = 167)					
Age (years)	3.936 ± 3.530	6.892 ± 3.734	−0.228	0.047	23.262	0.000*	0.796 (0.725–0.873)
Gender *n* (%)							
Boy	29 (46.8%)	107 (64.1%)	0.044	0.3	0.021	0.884	1.045 (0.580–1.882)
Girl	33 (53.2%)	60 (35.9%)					
Height (cm)	104.298 ± 30.270	125.889 ± 28.100	−0.026	0.006	20.907	0.000*	0.974 (0.964–0.985)
Bmi	15.336 ± 2.414	17.034 ± 4.376	−0.136	0.049	7.684	0.006*	0.873 (0.793–0.961)
Allergy history							
Not have	42 (67.8%)	148 (88.6%)	−0.997	0.379	6.917	0.009*	0.369 (0.176–0.776)
Have	20 (32.2%)	19 (11.4%)					
Percentage of eosinophils	4.382 ± 4.148	2.884 ± 2.217	0.159	0.049	10.53	0.001*	1.172 (1.065–1.290)
Absolute eosinophils	0.401 ± 0.508	0.208 ± 0.170	2.134	0.607	12.373	0.000*	8.452 (2.573–27.761)
Wear monitoring Time	185.823 ± 145.192	93.677 ± 51.091	0.015	0.003	27.333	0.000*	1.015 (1.009–1.021)

* Denotes *P* < 0.01.

#### 5.2.2 Screening variables using backward stepwise regression

Age, height, BMI, allergy history, eosinophil percentage, eosinophil absolute value, and wear time, which showed statistically significant differences in univariate analysis, were included in the multifactorial logistic regression analysis. The results indicated that allergy history, age, wear time, eosinophil percentage, and whether or not the children developed skin lesions were correlated (Table 4).

**Table 4:** Multifactor logistic regression analysis.

Variant	β-value	Standard error	Wald	*P*	Or value (95% CI)
History of allergies (1)	−1.099	0.475	5.365	0.021	0.333 (0.131–0.844)
Age (years)	−0.228	0.058	15.657	0.000*	0.796 (0.711–0.891)
Wear monitoring time	0.017	0.003	27.542	0.000*	1.017 (1.011–1.023)
Percentage of eosinophils	0.166	0.063	7.05	0.008*	1.181 (1.044–1.335)

* Denotes *P* < 0.01.

### 5.3 Comparison of the incidence of skin lesions in children

By optimizing the cardiac monitoring skin care program, the incidence of skin lesions in children significantly decreased. From September to December 2023, the intervention group had 29 cases of skin problems related to the use of cardiac monitoring electrode pads, with a skin lesion rate of 15.8%, meeting the intended goal. In the intervention group, where the percentage of allergies was higher than in the control group, there was a statistically significant improvement in skin redness and rash after electrode pad removal. Although the rate of breakage decreased, no statistically significant difference was observed for blisters or breakage. Regarding the skin damage score, a higher proportion of children in both groups had scores between 0 and 4, with a statistically significant difference between the two groups. However, no significant difference was found in higher scores, which may be attributed to the small sample size and will require further investigation in the future (Table 5).

**Table 5:** Comparison of skin damage between the Two groups of children [*n* (%)].

Variant	Control group (% cases)	Intervention group (% of cases)	Statistical value	*P*
Total cases (*n*)	229	183		
Redness of the skin	53 (32.8)	14 (4.2)	4.096	<0.001*
Rashes	29 (9.2)	10 (2.1)	2.361	0.0137
Blisters	7 (2.6)	3 (1.6)	0.928	0.3383
Breakages	4 (1.7)	2 (1.0)	0.549	0.5735
Skin damage score	148	51		
0–4	222 (96.9%)	181 (98.9%)	3.117	0.0014*
5–8	4 (1.8%)	0 (0.0%)	–	–
9–12	3 (1.3%)	2 (1.1%)	–	–

* Denotes *P* < 0.01.

## 6 Discussion

### 6.1 Integrated interpretation of risk factors and pathogenesis

The risk factors for MARSI identified in this study align with and expand the current international consensus (8). Notably, the negative protective effects of age and BMI (*β* = −0.23 to −0.28) highlight a bidirectional modulation of skin barrier function by developmental physiological features. On one hand, the thinner stratum corneum in infants and young children (∼9.9 μm vs. 20.3 μm in adolescents) (12) and the higher epidermal water loss rate (TEWL ≥25 g/m^2^/h) exacerbate the penetration damage of medical adhesive (13, 14). On the other hand, the cushioning effect of subcutaneous adipose tissue in overweight children (BMI ≥18 kg/m^2^) helps disperse the patch shear force.

The predictive value of eosinophilic granulocytes (EOS) is a key finding in this study. Children with an EOS percentage >5% have a 4.1-fold increased risk of MARSI (adjusted OR = 4.1). The mechanism may involve the following pathways: (i) Activated EOS release major basic proteins (MBPs), which disrupt keratinocyte intercellular junctions directly (15) (ii) EOS-derived IL-5 and CCL5 chemokines recruit Th2 cells, amplifying the local inflammatory cascade (16). This provides a molecular biological basis for stratified interventions based on dynamic monitoring of EOS.

### 6.2 Multidimensional efficacy mechanism of the layered care protocol

The clinical benefits of this protocol, which reduced the incidence of MARSI from 40.6% to 15.8%, were derived by three innovations:

(a) Material engineering optimization: The microporous structure of the silicone foam dressing (pore size 50–200 μm) reduces the shear force at the patch-skin interface by 62% (17), while maintaining a breathability of more than 90%, thus avoiding the risk of maceration caused by traditional hydrocolloid dressings.
(b) Time-paced interventions: The intermittent pause strategy (≥4 h off every 48 h) in high-risk children effectively breaks the vicious cycle of “adhesive stress accumulation - stratum corneum dehydration”.
(c) Evidence-based skin pre-treatment: Allergy-free emollient cream (Cetaphil) repairs lipid bilayers by supplementing ceramides (≥3%), increasing skin hydration by 58.3%, as demonstrated by Raman spectroscopy (18, 19), significantly reducing mechanical damage during adhesive attachment.

### 6.3 PDCA system-driven quality of care transformation

The “Plan-Do-Check-Act (PDCA)” quality control closed-loop system implemented by our center effectively addressed three major shortcomings in the prevention and control of previous MARSIs: Cognitive disconnection, Operational heterogeneity and data fragmentation. Through case simulation training, nurses’ recognition accuracy of high-risk signs (e.g., EOS absolute value >0.5 × 10^9^/L) increased from 64% to 92%; the intelligent timing system reduced the electrode patch replacement time deviation from ±3.2 h to ±0.5 h (*P* < 0.001); The nursing management platform enabled real-time capture and root cause analysis of MARSI events, reducing the quality improvement cycle from 14 days to 7 days. These improvements align with the core elements of the JCI accreditation system, particularly the “accurate control of high-risk technologies” (20) and provide a replicable quality control template for promoting standardized management of children's medical adhesives.

### 6.4 Research limitations and translational medicine perspectives

Despite the significant results, the following limitations exist:

(a) Population specificity: The study focused on postoperative children with post-coronary artery disease, whose systemic inflammatory status may overestimate the predictive value of EOS. Validation in cardiology and general pediatrics cohorts is required.
(b) Cost-effectiveness blindness: Silicone foam dressings increase the cost of a single episode of care. In the future, biodegradable biomucoadhesives should be developed to balance efficacy and cost.
(c) Lack of longitudinal data: The study lacked a 1-month post-discharge follow-up to assess delayed impairment due to inadequate home care.

It is recommended that follow-up studies focus on:

(d) Population expansion studies: Verify the universal applicability of EOS predictive value across diverse populations, including respiratory department (asthma) and neonatal department (preterm infants).
(e) Threshold optimization: Determine the age-stratified cutoff values of EOS through ROC curve analysis, and explore tolerance threshold variations among different pediatric age groups during ECG monitoring with skin electrodes.
(f) Control of confounding factors: Establish a multivariate adjustment model to eliminate the interference from surgical stress and glucocorticoid use on EOS levels.
(g) Construction of a full-cycle care pathway: Integrate precise in-hospital prevention and control with continuous community or home-based care, and provide an executable operational model for pediatric patients undergoing home-based ECG monitoring.

In addition, cardiac monitoring operations are expected to break through in the following areas:

(a) New material development: The development and use of new removable adhesives (e.g., those that can significantly reduce the risk of mechanical injury), and pH-sensitive tapes or adhesives, which change color to warn of changes in the skin microenvironmental pH, will enable individualized and precise monitoring (21).
(b) AI-based skin monitoring system: The development of an app for early MARSI identification can facilitate individualized and precise data analysis, utilizing picture comparison, data analysis, etc., to detect early MARSI and achieve effective prevention and treatment (22).

## Data Availability

The raw data supporting the conclusions of this article will be made available by the authors, without undue reservation.
